# Muscle Activation Differs between Three Different Knee Joint-Angle Positions during a Maximal Isometric Back Squat Exercise

**DOI:** 10.1155/2016/3846123

**Published:** 2016-07-18

**Authors:** Paulo Henrique Marchetti, Josinaldo Jarbas da Silva, Brad Jon Schoenfeld, Priscyla Silva Monteiro Nardi, Silvio Luis Pecoraro, Julia Maria D'Andréa Greve, Erin Hartigan

**Affiliations:** ^1^Graduate Program in Science of Human Movement, College of Health Science (FACIS), Methodist University of Piracicaba, 13423-070 Piracicaba, SP, Brazil; ^2^Institute of Orthopedics and Traumatology, School of Medicine, University of São Paulo, Laboratory of Kinesiology, 05403-000 São Paulo, SP, Brazil; ^3^Department of Health Sciences, Program of Exercise Science, CUNY Lehman College, Bronx, NY 10468, USA; ^4^Department of Physical Therapy, University of New England, Portland, ME 04103, USA

## Abstract

The purpose of this study was to compare muscle activation of the lower limb muscles when performing a maximal isometric back squat exercise over three different positions. Fifteen young, healthy, resistance-trained men performed an isometric back squat at three knee joint angles (20°, 90°, and 140°) in a randomized, counterbalanced fashion. Surface electromyography was used to measure muscle activation of the vastus lateralis (VL), vastus medialis (VM), rectus femoris (RF), biceps femoris (BF), semitendinosus (ST), and gluteus maximus (GM). In general, muscle activity was the highest at 90° for the three quadriceps muscles, yet differences in muscle activation between knee angles were muscle specific. Activity of the GM was significantly greater at 20° and 90° compared to 140°. The BF and ST displayed similar activation at all joint angles. In conclusion, knee position alters muscles activation of the quadriceps and gluteus maximus muscles. An isometric back squat at 90° generates the highest overall muscle activation, yet an isometric back squat at 140° generates the lowest overall muscle activation of the VL and GM only.

## 1. Introduction

The squat is one of the most frequently used exercises in the field of strength and conditioning. The squat is an exercise that increases hip and knee extensor muscle strength which then indirectly improves the quality of life in athletic and nonathletic populations [1]. The squat exercise utilizes muscles with different morphology (monoarticular and biarticular). Muscle forces also vary depending on joint positions (moment arm, length-tension relationship), whether the muscle acts as a prime mover or stabilizer, and whether the task is dynamic or static. Though evidence suggests that architecture, position, and function drive muscle performance during the squat, little is known about the neuromuscular changes that occur from a muscle activation standpoint. Elucidating how muscle activation patterns change in the monoarticular and biarticular knee and hip extensors during squatting at different knee angles would thus enhance our understanding of how one could capitalize on maximizing muscle activation and the best position to specific evaluations and sEMG normalization. This would be a first step to then apply the knowledge during exercise prescription that includes the squat.

Considering that the squat exercise is a multijoint task, a large number of muscle groups can be activated simultaneously in a more complex way. Several studies have shown that manipulating features of the squat exercise resulted in altered muscle activity. These manipulations include changes in foot position [2, 3], barbell position [4], stability of the surface on which the exercise is performed [5–9], different levels of intensity of load [10], range of motion [10–12], and different equipment [13].

As a multijoint exercise, the knee extensors (e.g., rectus femoris, RF; vastus lateralis, VL; and vastus medialis, VM) and hip extensors (e.g., gluteus maximus, GM; biceps femoris, BF; and semitendinosus, ST) are considered to be the prime movers during the squat exercise, with other muscles acting in a secondary capacity [1, 11, 14]. Caterisano et al. [11] measured the relative contributions of GM, BF, VM, and VL muscles of ten experienced lifters while performing dynamics squats at 3 depths (partial squat (the angle between the femur and the tibia was ~2.36 rad at the knee joint), parallel squat (the angle between the femur and the tibia was ~1.57 rad at the knee joint), and full-depth squat (the angle between the femur and the tibia was ~0.79 rad at the knee joint)), using 100–125% of body weight as resistance. The results suggested that the GM was most active, rather than the BF, the VM, or the VL, during a concentric contraction as squat depth increases. On the contrary, Robertson et al. [12] determined that the GM displayed a reduced activity level at maximum squat depth. Robertson et al. [12] also showed that the biarticular muscles functioned mainly as stabilizers of the ankle, knee, and hip joints by working eccentrically to control descent or transferring energy among the segments during ascent. Whether monoarticular and biarticular hip and knee extensors have different muscle activation during an isometric squat and whether activation changes during different knee angles are unclear. Consequently, the rationale of the present study was to evaluate, indirectly, the muscle activation in different mechanical positions related to differences in the joint-angle-torque diagram and the sticking region effect in all three joint angles (20°, 90°, and 140°).

Finally, differences in muscle activity during dynamic and isometric squat exercise have received less attention in the physical education and rehabilitation area. Others have shown the isometric squat (90° and 120° of knee join position) as a reliable test to provide an indicator of changes in dynamic strength (1-repetition maximum barbell back squat, 1RM) and power performance [15, 16]; however, whether muscle activity changes as an isometric squat is manipulated is unknown. Although motor units are recruited differently during dynamic movements, they generate the same relative force/torque during a static contraction [17]. Despite inherent neural and mechanical differences between isometric and dynamic contractions, the isometric squat exercise performed in different knee joint angles may be used to understand changes in muscle activation patterns without confounding any other external effects such as the stretch-shortening cycle from dynamic movements [18]. Therefore, the purpose of this study was to evaluate the maximal isometric muscle activation of the lower limbs during three different knee joint-angle positions in the back squat exercise.

## 2. Methods

### 2.1. Subjects

We collected the peak amplitude of the root mean square (RMS) from VL sEMG data during a pilot study to drive this power analysis. Based on a statistical power analysis derived from these data (RMS VL EMG), it was determined that twelve subjects would be necessary to achieve an alpha level of 0.05, effect size of 1.41, and a power (1 − *β*) of 0.80 [19]. Therefore, we recruited fifteen young, healthy, resistance-trained men (age: 30 ± 7 years, height: 174 ± 6 cm, and total body mass: 76 ± 9 kg, with 5 ± 1 years of experience on the back squat exercise) to participate in this study. Subjects had no previous lower back injury, no surgery on the lower extremities, and no history of injury with residual symptoms (pain, “giving-away” sensations) in the lower limbs within the last year. This study was approved by the university research ethics committee and all subjects read and signed an informed consent document.

### 2.2. Procedures

Prior to data collection, subjects were asked to identify their preferred leg for kicking a ball, which was then considered their dominant leg [20]. All subjects were right-leg dominant. Volunteers attended one session in the laboratory, and they reported to have refrained from performing any lower body exercise other than activities of daily living for at least 48 hours prior to testing. Subjects performed a 5-minute cycle warm-up and a familiarization session with all isometric conditions. The familiarization session was performed in all joint angles used during the experimental procedure (20°, 90°, and 140°) for 1 set of 3 seconds each. After the warm-up and familiarization, all subjects performed three trials of 10-second maximal isometric contractions against a locked smith machine under three different knee joint-angle positions in a randomized, counterbalanced order: back squat at 20 degrees (20°); back squat at 90 degrees (90°); and back squat at 140 degrees (140°). The knee joint-angle positions were evaluated by a goniometer (Plastic 12′′ Goniometer 360 Degree ISOM), and, for all angles, full knee extension was considered the “zero” position. The subjects' feet were always positioned at hip width and vertically aligned with the barbell position. The barbell was positioned on the shoulders (high-bar position) for all subjects and experimental conditions. A rest period of 15 minutes was provided between conditions with 3 minutes afforded between sets. All measures were performed at the same hour of the day, between 9 and 12 AM, and by the same researcher.

### 2.3. Measures

#### 2.3.1. Surface Electromyography (sEMG)

The subjects' hair was shaved at the site of electrode placement and the skin was cleaned with alcohol before the sEMG electrode was affixed. Bipolar active disposable dual Ag/AgCl snap electrodes were used which were 1 cm in diameter for each circular conductive area with 2 cm center-to-center spacing. Electrodes were placed on the dominant limb along the axes of the muscle fibers, according to the SENIAM/ISEKI protocol [21]: gluteus maximus (GM) at 50% of the distance between the sacral vertebrae and the greater trochanter; vastus lateralis (VL) at 2/3 of the distance between the anterior spine iliac and the superior aspect of the lateral side of the patella; rectus femoris (RF) at 50% on the line from the anterior spine iliac to the superior part of patella; vastus medialis (VM) at 80% on the line between the anterior spine iliac superior and the joint space in front of the anterior border of the medial ligament; biceps femoris (BF) at 50% on the line between the ischial tuberosity and the lateral epicondyle of the tibia; and semitendinosus (ST) at 50% on the line between the ischial tuberosity and the medial epicondyle of the tibia. The sEMG signals were recorded by an electromyographic acquisition system (EMG832C, EMG System do Brasil, São José dos Campos, Brazil) with a sampling rate of 2000 Hz using a commercially designed software program (EMG System do Brasil, São José dos Campos, Brazil). EMG activity was amplified (bipolar differential amplifier, input impedance = 2 MΩ, common mode rejection ratio > 100 dB min (60 Hz), gain × 20, noise > 5 *μ*V) and converted from an analog to digital signal (12 bits). A ground electrode was placed on the right clavicle. EMG signals collected during all conditions were normalized to a maximum voluntary isometric contraction (MVIC) against fixed strap resistance. Then, two trials of five-second MVICs were performed for each muscle with one-minute rest between actions for the dominant leg. The first MVIC was performed to familiarize the participant with the procedure. For GM MVIC, subjects were in the prone position with their knee flexed at 90° and resistance placed on the distal region of the thigh with the pelvis stabilized. For VL, VM, and RF MVICs, subjects were seated with their knee flexed at 90° and resistance placed on the distal tibia. For BF and ST MVICs, subjects were seated with their knee flexed at 90° and resistance placed on the distal tibia. Verbal encouragement was given during all MVICs. The order of MVICs was counterbalanced to avoid any potential neuromuscular fatigue.

### 2.4. Data Analyses

All sEMG data were analyzed with customized Matlab routine (MathWorks Inc., USA). The digitized sEMG data were band-pass filtered at 20–400 Hz using a fourth-order Butterworth filter with zero lag. For muscle activation time domain analysis, RMS (150 ms moving window) was calculated during the MVIC. Isometric back squat data was then normalized to the RMS peak of the two peak MVICs, the first second was removed from RMS normalized, and the following 3 seconds of each trial were integrated (iEMG).

### 2.5. Statistical Analyses

The normality and homogeneity of variances within the data were confirmed with the Shapiro-Wilk and Levene's tests, respectively. To test differences for each muscle activity (iEMG), repeated measures ANOVAs were used. Post hoc comparisons were performed with the* Bonferroni* test. Cohen's formula for effect size (*d*) was calculated, and the results were based on the following criteria: <0.35 trivial effect; 0.35–0.80 small effect; 0.80–1.50 moderate effect; and >1.5 large effect, for recreationally trained subjects [22]. Interrater reliability was assessed for the researcher who positioned and evaluated iEMG tracings for all muscles and conditions. Reliability was operationalized using the following criteria: <0.4 poor; 0.4–<0.75 satisfactory; ≥0.75 excellent [23]. The ICCs ranged between 0.91 and 0.99 (excellent) for all iEMG data. An alpha of 5% was used to determine statistical significance.

## 3. Results

There was a significant main effect of VL (*P* < 0.001), VM (*P* = 0.030), RF (*P* = 0.018), and GM (*P* < 0.001) for muscle activity during three different knee joint-angle positions (20°, 90°, and 140°) in the isometric back squat.

**Figure 1:**
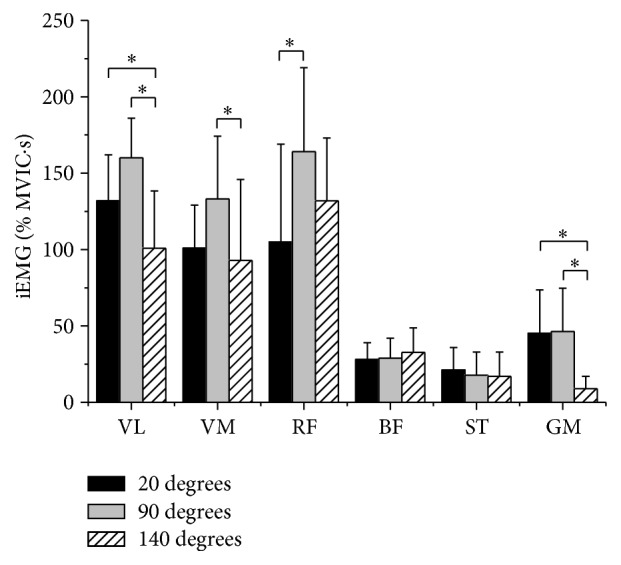
Mean and standard deviation of iEMG in three different knee joint-angle positions. ^*∗*^Significant differences, *P* < 0.05.

The VL activity was significantly less in 140° than 20° (*P* = 0.027, Δ% = 24.4) and 90° (*P* < 0.001, Δ% = 37.5). The VM activity was significantly less in 140° than 90° (*P* = 0.036, Δ% = 30). The RF activity was significantly less in 20° than 90° (*P* = 0.015, Δ% = 36). The GM activity was significantly less in 140° than 90° (*P* < 0.001, Δ% = 80.4) and 20° (*P* < 0.001, Δ% = 80) (Table 1 and Figure 1).

## 4. Discussion

The purpose of this study was to evaluate the maximal isometric muscle activation of the lower limbs during three different knee joint-angle positions in the back squat exercise. The architecture, position, and function influence muscle forces during the squat; however, little is known about the neuromuscular changes that occur from a muscle activation standpoint. The primary finding of this investigation was that, during isometric squatting, a position of 90° of knee joint angle demonstrated the overall highest muscle activation of the quadriceps and gluteus maximus, whereas the 140° knee joint-angle position presented the lowest muscle activation values for almost all muscles that act as prime movers. Interestingly, the activation of the hamstring did not differ among knee joint-angle positions and the three quadriceps muscles responded differently as the knee went from a relatively extended position to a more flexed position.

Given the close chain nature of the squat, as the knee joint changes position, the hip joint angles also change positions. Consequently, the squat exercise simultaneously utilizes several muscles with different morphologies (monoarticular and biarticular) in a manner that produces “muscle coordination” [24]. A multijoint task to strengthen the knee and hip extensors is more complex for the neuromuscular system as two joints work in concert to achieve the task [12]. Also, since some muscles cross more than one joint, the complexity increases compared to open chain terminal knee extension or isolated hip extension exercise [12]. During the squat exercise, there are several biarticular muscles interacting including the hamstrings and RF [1]. Biarticular muscles such as RF, BF, and ST have intermediate activation when the muscles have agonistic action at one joint and antagonistic action at the other joint; this is in contrast with the high activation seen when a biarticular muscle works as an agonist for both joints simultaneously [24]. Lombard [25] suggested that biarticular muscles of the lower extremity act in a “paradoxical” fashion when the movement is constrained or controlled (named* Lombard's paradox*); it is observed when RF, BF, and ST contract concurrently when rising from a chair. The extension seen from both the hip and the knee is the result of the differential moment arms of the two muscles at each joint. The present results showed low muscle activation for BF and ST in all knee positions, probably because these muscles act more like a joint stabilizer at the knee and a prime mover at the hip. Both BF and ST have the longer moment arm at the hip thereby creating a hip extensor moment. Thus, the BF and ST muscles allow for the extension of both the knee and the hip [12]. Since the RF has a greater moment arm across the knee, due to the patella, it creates an extensor moment at the knee joint. Considering the present results, the RF showed higher muscle activation at 90° when compared to 20°; however, it was similar to 140° of knee angle. This may represent a higher effect on muscle activation during the initial phase of the squat movement (between 20° and 90°) than after 90° since the muscle activation did not change.

On the other hand, monoarticular muscles act on one specific joint. During the squat exercise, several monoarticular muscles contribute to movement including the soleus, vasti (lateralis, medialis, and intermedius), and GM [1]. The present results showed that muscle activation for all monoarticular muscles (e.g., VM, VL, and GM) did not differ between 20° and 90°. Additionally, the highest muscle activation was observed at 90° when compared to 20° and 140°; on the other hand, 140° presented the lowest activation for VL and GM muscles. Interestingly, the VM behaved differently from the other monoarticular muscles, even the VL, as the muscle activation of the VM did not differ between the 20- and 140-degree knee joint angles.

Usually, when monoarticular muscles perform as agonists, the activation increases as the joint moment increases [24]. Additionally, monoarticular muscles are affected by the sticking region which is considered a poor mechanical force position in which the mechanical advantage of the muscles involved is such that their capacity to exert force is reduced and where the lifter experiences difficulty in exerting force against the external load [26–30]. Cardinale et al. [31] displayed that the higher muscle activation during the squat exercise occurs at 90° of knee joint-angle position, which is considered the sticking region. The present results support this finding for all monoarticular muscles analyzed (VL, VM, and GM). Our findings support this theory as all monoarticular muscles presented lower values of activation at 140° of knee joint-angle position when compared to 90°. In this specific position (at 140°), it is feasible to speculate that changes in muscle length modify muscle contractile abilities and, in turn, modify sEMG-force and sEMG-moment relationships [18, 24]. Alternatively, afferent signals from muscles could decrease motoneuronal firing frequency (i.e., Golgi tendon reflex) during isometric contractions when the muscle fibers are in an elongated position [17].

Others have also investigated muscle activation during the squat by comparing different knee joint angles, yet previous studies compared knee positions during a dynamic squat, not an isometric squat. Caterisano et al. [11] measured the relative contributions of GM, BF, VM, and VL muscles of ten experienced lifters while performing dynamics squats at 3 depths (partial squat (the angle between the femur and the tibia was ~2.36 rad at the knee joint), parallel squat (the angle between the femur and the tibia was ~1.57 rad at the knee joint), and full-depth squat (the angle between the femur and the tibia was ~0.79 rad at the knee joint)), using 100–125% of body weight as resistance. Caterisano et al. [11] found that, during the concentric phase of the dynamic squat, the GM activation was higher during full-depth (35.4%) squat compared to the partial (16.9%) and parallel (28.0%) squat exercise and that the BF, the VM, and the VL did not change. The results suggested that the GM, rather than the BF, the VM, or the VL, becomes more active in concentric contraction as squat depth increases. Others have also shown superior muscular hypertrophy when squatting throughout a full versus a partial range of motion [32, 33]. Our findings of less muscle activation at 140° do not support Bloomquist and colleagues' findings. The greater cross-sectional area of the muscles found by Bloomquist et al. [32] may be more related to time under tension than the muscle activation. However, without muscle activation data, this remains speculative. In opposition, when our subjects performed an isometric squat in different positions, the GM activity was the highest in the 90-degree position, not the deeper knee flexion position. Perhaps the change in 100–125% body weight load during the dynamic trials and our maximum isometric load in all three conditions influence the lack of agreement between the studies. Similar to our results, Robertson et al. [12] reported that the GM muscle activity level was reduced at maximum full (deep-knee) squat depth. Robertson et al. [12] also concluded that the biarticular muscles (BF, ST, and RF) functioned mainly as stabilizers of the knee and hip joints during the eccentric and concentric phases of a dynamic squat. The authors presumed that the reduced GM activity level at maximum squat depth was because the GM was not needed to maintain stability or perhaps it permitted an extra degree of hip flexion that created a deeper countermovement immediately before the ascent phase. From an activation standpoint, our findings suggest a diminished benefit from squatting beyond 90°. The reason for these seemingly contradictory findings among studies remains to be elucidated. Investigations comparing muscle activity during isometric and dynamic squatting are needed.

A limitation of this study includes the use of healthy, well-trained men only, and, therefore, our findings are not generalizable to other conditions, populations, or women. We also have a small sample size; thus, this study may be underpowered to identify differences between knee joint positions. We did not control for hip angles to create a more realistic squat performance.

## 5. Conclusion

Knee position alters muscles activation of the quadriceps and gluteus maximus muscles. An isometric back squat at 90° generates the highest overall muscle activation, yet an isometric back squat at 140° generates the lowest overall muscle activation of the VL and GM only. Knee angle did not affect muscle activation of the hamstrings. Thus, we recommend performing an isometric squat at 90° to maximize neuromuscular recruitment of the knee and hip extensors.

## Figures and Tables

**Table 1:** Effect size (*d*) comparisons for iEMG between knee joint-angle positions.

Angle	VL	VM	RF	BF	ST	GM
20° × 90°	0.99	0.91	0.98	0.08	0.20	0.03
90° × 140°	1.87	0.87	0.65	0.27	0.05	1.81
20° × 140°	0.95	0.18	0.50	0.36	0.24	1.76
